# Supplementary material for: Topologically frustrated ionisation in a water-ammonia ice mixture
Source: Nat Commun. 2017 Oct 20;8:1065. doi: 10.1038/s41467-017-01132-z (PMC5648802; doi:10.1038/s41467-017-01132-z)
Supplement: Supplementary file 1 — Supplementary Information [file 41467_2017_1132_MOESM1_ESM.pdf]

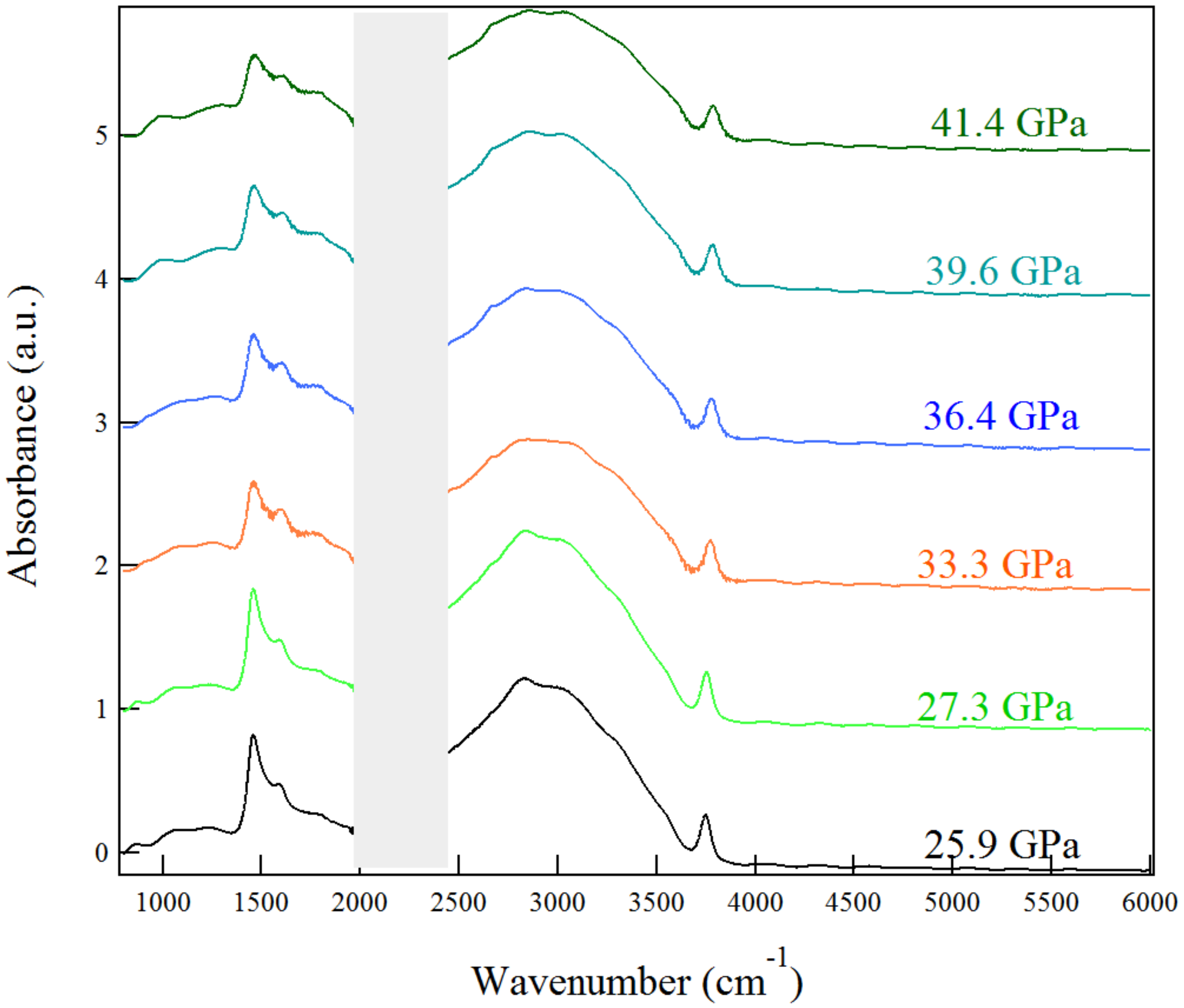

Supplementary Figure 1. Evolution of the infrared absorption spectrum of AMH (Ammonia Monohydrate) with pressure at ambient temperature.

The frequency window from 2000 to 2400 cm^{-1} is obscured by the strong absorption band of the diamond anvils. The spectra are offset for clarity and the pressure is indicated in GPa.

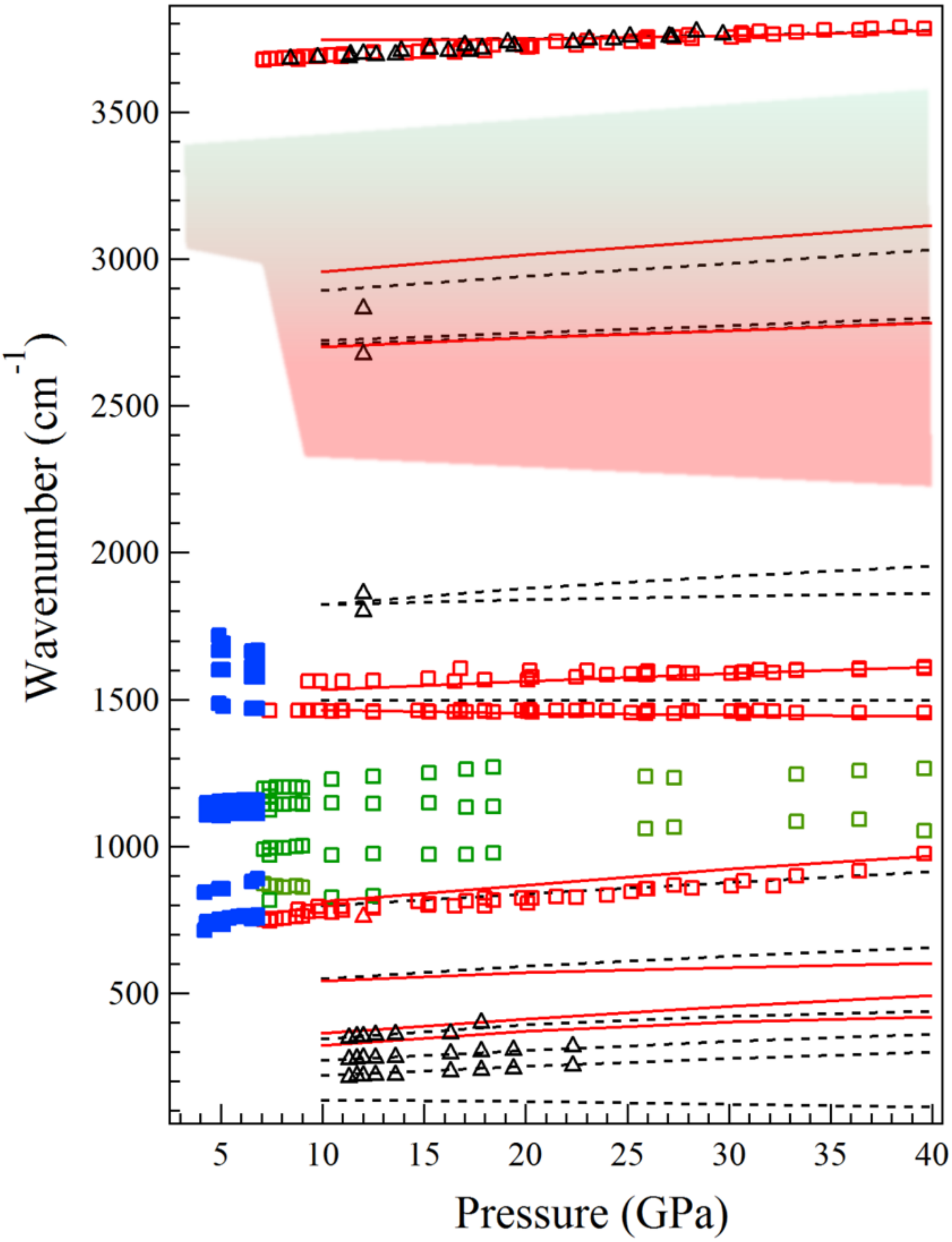

Supplementary Figure 2. Pressure evolution of the Raman and infrared band wavenumbers of AMH at ambient temperature.

Experimental data are represented with symbols. The continuous and discontinuous lines respectively represent the calculated wavenumbers of the infrared and Raman modes in the *P4/nmm* structure. Red open squares indicate IR modes compatible with the predicted ionic *P4/nmm* structure while green open squares correspond to the molecular NH_3 and H_2O IR modes above 7.4 GPa. The red/green shaded region correspond to the broad IR absorption in the stretching region of NH_4^+, OH^- and NH_3. Solid blue squares show IR modes below the phase transition at 7.4 GPa, where modes from the ionic species disappear. The Raman experimental data are shown by open triangles.

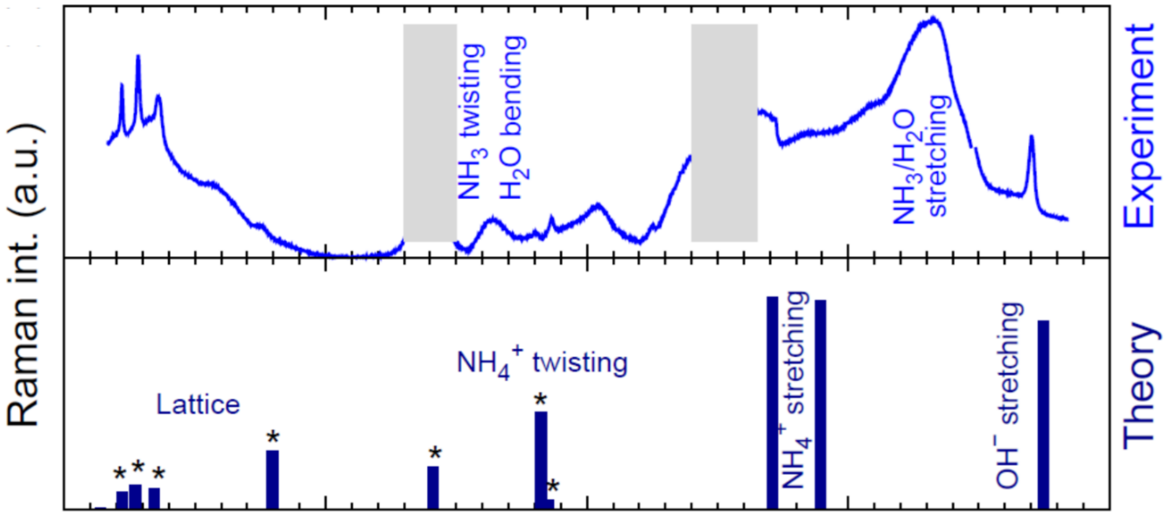

Supplementary Figure 3. Raman spectrum of AMH

The upper plot shows the experimental Raman spectrum collected at 12 GPa and ambient temperature from a different sample of AMH than shown in the main text (Fig. 2). The peaks from the ionic *P4/nmm* structure are weaker in this sample. The frequency windows 1300-1400 cm^{-1} and 2200-2600 cm^{-1} are greyed as they are dominated by, respectively, the first and second order Raman signal from the diamond anvils The theoretical Raman spectra for the *P4/nmm* structure is shown in the lower panel. For visibility, Raman intensities have been multiplied by a factor 30 for modes labeled with a * symbol.

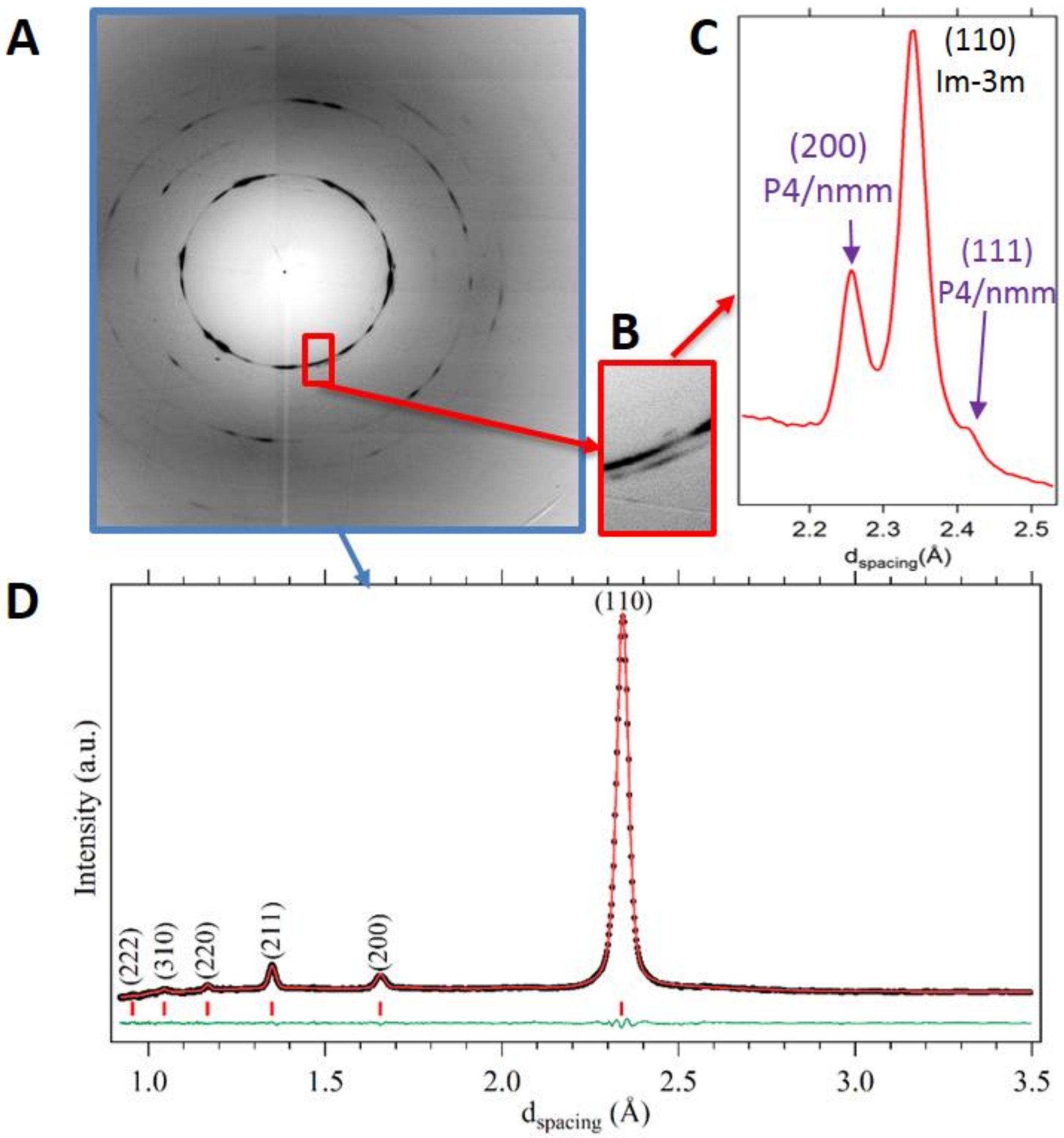

Supplementary Figure 4. X-ray diffraction pattern of AMH

Panel **A** shows the x-ray diffraction bi-dimensional image collected at 12 GPa and 300 K from the sample whose Raman spectrum is presented in Supplementary Fig. 3. Consistent with the Raman spectrum, the Bragg peaks from the *P4/nmm* structure are less intense in this sample, and the diffraction pattern is dominated by the *Im-3m* phase. Panel **B** shows an enlarged view of the red box to show the two additional partial rings on each side of the (110) ring of *Im-3m* and indexed as the (200) and (111) peaks of the *P4/nmm* structure. Panel **C** shows the one-dimensional patterns obtained by integrating the zoomed region. The pattern obtained by the integration of the complete image is shown in panel **D**. The two peaks of *P4/nmm* barely stand out from the background in this integration. In **D**, the symbols are experimental data, the red solid line is a full profile Rietveld refinements using the *Im-3m* model of Loveday *et al*. (*Phys. Rev. Lett*., 83, 4329 (1999)). The green line shows the difference between observed and calculated profiles. Sticks show the positions of Bragg reflections of *Im-3m*.

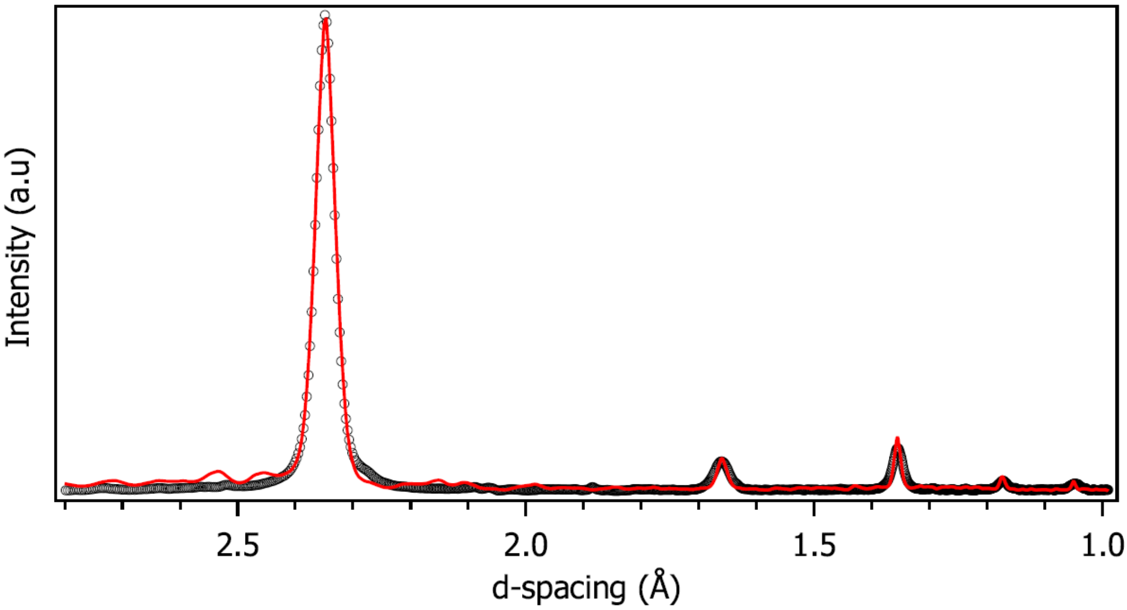

Supplementary Figure 5. X-ray diffraction pattern of the disorderd ionico-molecular alloy

The red line is the simulated x-ray diffraction pattern of the *Im-3m* phase (6x6x6 simulation box) after relaxation at 0 K and containing 13% of ionic species. The symbols show the (background-subtracted) experimental x-ray pattern at 12 GPa, 295 K (symbols) from the same sample as in Supplementary Fig. 3 and Supplementary Fig. 4. The *x* scale of the simulated pattern has been multiplied by 0.998 to account for the density difference, and the intensity adjusted to scale with the main (110) peak. The Bragg peaks were modeled by pseudo-Voigt profiles.

Atom	Wickoff positions	x	y	z
**H**	8i	0.2500	0.5687	0.8029
**H**	2c	0.2500	0.2500	0.2775
**N**	2a	0.7500	0.2500	0.0000
**O**	2c	0.2500	0.2500	0.6013

Supplementary Table 1. Calculated P4/nmm structure at 12 GPa.

Atomic coordinates and Wyckoff positions of the calculated P4/nmm structure at 12 GPa. The calculated lattice parameters are a=4.84 Å and c=3.00 Å.
